# Altered metabolic-functional coupling in the epileptogenic network could predict surgical outcomes of mesial temporal lobe epilepsy

**DOI:** 10.3389/fnins.2023.1165982

**Published:** 2023-06-08

**Authors:** Siyu Yuan, Hui Huang, Bingyang Cai, Jiwei Li, Miao Zhang, Jie Luo

**Affiliations:** ^1^School of Biomedical Engineering, Shanghai Jiao Tong University, Shanghai, China; ^2^Department of Nuclear Medicine, Ruijin Hospital, Shanghai Jiao Tong University School of Medicine, Shanghai, China

**Keywords:** MTLE, PET/MR, standardized uptake value ratio (SUVR), fractional amplitude of low-frequency fluctuation (fALFF), default mode network (DMN), epileptogenic network

## Abstract

**Objective:**

To investigate the relationship between glucose metabolism and functional activity in the epileptogenic network of patients with mesial temporal lobe epilepsy (MTLE) and to determine whether this relationship is associated with surgical outcomes.

**Methods:**

^18^F-FDG PET and resting-state functional MRI (rs-fMRI) scans were performed on a hybrid PET/MR scanner in 38 MTLE patients with hippocampal sclerosis (MR-HS), 35 MR-negative patients and 34 healthy controls (HC). Glucose metabolism was measured using ^18^F-FDG PET standardized uptake value ratio (SUVR) relative to cerebellum; Functional activity was obtained by fractional amplitude of low-frequency fluctuation (fALFF). The betweenness centrality (BC) of metabolic covariance network and functional network were calculated using graph theoretical analysis. Differences in SUVR, fALFF, BC and the spatial voxel-wise SUVR-fALFF couplings of the epileptogenic network, consisting of default mode network (DMN) and thalamus, were evaluated by Mann-Whitney U test (using the false discovery rate [FDR] for multiple comparison correction). The top ten SUVR-fALFF couplings were selected by Fisher score to predict surgical outcomes using logistic regression model.

**Results:**

The results showed decreased SUVR-fALFF coupling in the bilateral middle frontal gyrus (*P*_*FDR*_ = 0.0230, *P*_*FDR*_ = 0.0296) in MR-HS patients compared to healthy controls. Coupling in the ipsilateral hippocampus was marginally increased (*P*_*FDR*_ = 0.0802) in MR-HS patients along with decreased BC of metabolic covariance network and functional network (*P*_*FDR*_ = 0.0152; *P*_*FDR*_ = 0.0429). With Fisher score ranking, the top ten SUVR-fALFF couplings in regions from DMN and thalamic subnuclei could predict surgical outcomes with the best performance being a combination of ten SUVR-fALFF couplings with an AUC of 0.914.

**Conclusion:**

These findings suggest that the altered neuroenergetic coupling in the epileptogenic network is associated with surgical outcomes of MTLE patients, which may provide insight into their pathogenesis and help with preoperative evaluation.

## Highlights

- Both metabolic network and functional network are altered in mesial temporal lobe epilepsy (MTLE) patients.
- Metabolic-functional coupling is altered in the epileptogenic network of MTLE.
- SUVR-fALFF coupling in DMN and thalamic subnuclei are predictive of surgical outcomes.

## 1. Introduction

Mesial temporal lobe epilepsy (MTLE) is the most common type of drug-resistant epilepsy (Engel et al., 2012), characterized by abnormal neuronal discharge that results in repeated seizures arising from hippocampus and related mesial temporal lobe structures, which also play a key role in seizures propagation, and hippocampal sclerosis (HS) represents the most common pathology underlying MTLE (Bullmore and Sporns, 2009). Although anterior temporal lobectomy (ATL) is widely accepted as an effective surgical strategy for MTLE patients, ~40% of them have a poor prognosis 2 years after surgery (Liu et al., 2018). Emerging evidence indicates that MTLE is a network-based disease that exhibits widespread abnormalities affecting networks such as the default mode network (DMN) (Pittau et al., 2012; Chassoux et al., 2016; Englot et al., 2017; Tsuda et al., 2018; Zanao et al., 2021). Thalamus, an integrative hub for functional brain networks (Hwang et al., 2017), plays an important role in the process of seizure spread in MTLE (Zhang and Bertram, 2002), and particularly medial thalamic nuclei are involved in the limbic seizure circuitry (Guye et al., 2006; Bertram et al., 2008).

The hybrid PET/MR allows simultaneous measurement of ^18^F-FDG PET and functional MRI (fMRI), which gives us the opportunity of studying metabolic-functional coupling of the human brain. A previous study in healthy volunteers has found that changes in glucose metabolism were related to changes in rs-fMRI fluctuations (Tomasi et al., 2013). Another healthy volunteer research identified a significant spatial correlation between standardized uptake value ratio (SUVR) and fMRI metrics across the gray matter and DMN (Aiello et al., 2015). Further study demonstrated that brain-wide correlations between SUVR and amplitude of low-frequency fluctuation (ALFF) were significantly lower in TLE patients (Nugent et al., 2015). ALFF reflects the amplitude of spontaneous neural activities and fractional ALFF (fALFF) is demonstrated to be a more robust index than ALFF (Zang et al., 2007; Zou et al., 2008). Wang et al. (2020) reported that the coupling between SUVR and fALFF across gray matter was higher in patients with MR-HS compared to healthy controls and higher in patients who had bad outcomes after surgery than good ones. However, it is unclear whether the coupling within local epileptogenic network is disturbed in MTLE patients.

Since increasing evidence suggests that MTLE is a network disease and graph theory provides a promising method to describe topological and organizational properties of complex network (Bullmore and Sporns, 2009), more and more studies apply graph theoretical analysis with fMRI data and FDG PET data to investigate the abnormalities of whole brain network and epileptogenic networks in MTLE. A MR-negative MTLE study demonstrated that MR-negative was associated with impaired interictal functional connectivity (FC) of the ipsilateral temporal cortex, measured by betweenness centrality (BC) in graph theoretical analysis (Vaughan et al., 2016). Zhou et al. (2019) indicated that patients with TLE showed a functional disrupted topological reorganization within the DMN, characterized by decreased degree centrality of some brain regions in DMN. In addition, a previous investigation provided another view of evidence that MTLE was a system neurological disorder with disrupted networks, using hippocampus-based and whole-brain metabolic covariance networks (Wang et al., 2019). We hypothesized that the coupling between FDG SUVR and fALFF might be altered in epileptogenic network of MTLE patients.

In this study, we investigated the changes in glucose uptake, functional activity, metabolic and functional network properties and SUVR-fALFF couplings in the epileptogenic network of MTLE using simultaneous PET/MR, and further evaluated whether the couplings could predict surgical outcomes.

## 2. Materials and methods

### 2.1. Participants

In this IRB-approved study, we recruited 97 refractory unilateral mesial temporal lobe epilepsy patients between August 2018 and November 2021, and 32 healthy controls (HC). The inclusion criteria of patients are as follows: (1) diagnosis of temporal lobe epilepsy based on International League Against Epilepsy (ILAE) criteria (Blumcke et al., 2013); (2) MRI either normal or disclosed patterns suggestive of HS confirmed by histopathology. The exclusion criteria include (1) other neurological system lesion or other psychiatric disorders; (2) bilateral hippocampal sclerosis. For all patients, a comprehensive set of clinical evaluation including neurologic history, diagnostic MRI, FDG PET and scalp video-electroencephalography (EEG) recordings were collected.

**Figure 1:**
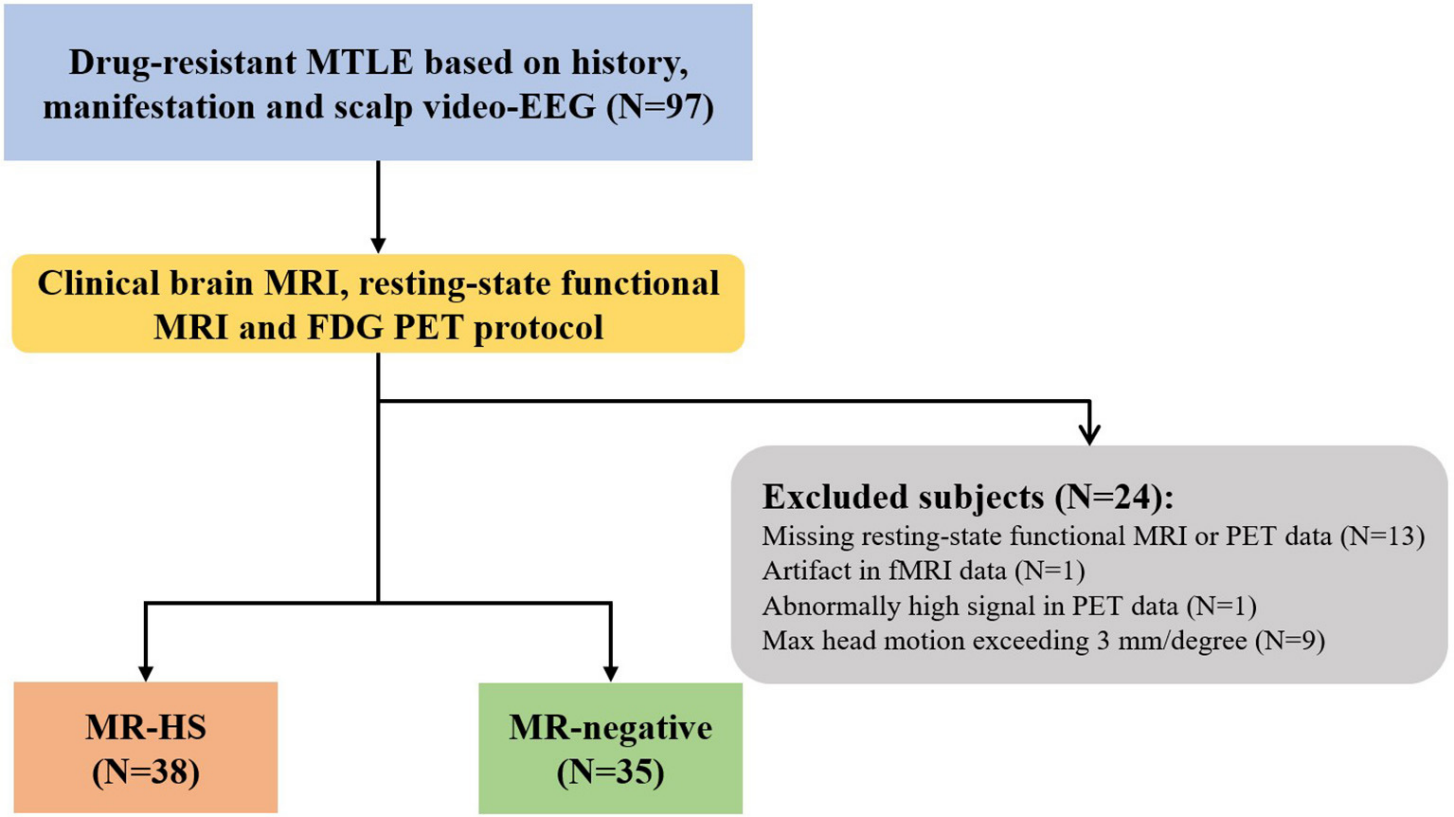
The flowchart of patient inclusion. MTLE, mesial temporal lobe epilepsy; HS, hippocampal sclerosis.

Twenty-four patients were further excluded due to incomplete or low-quality data: missing resting-state functional MRI or PET data (*N* = 13), artifact in rs-fMRI data (*N* = 1), abnormally high PET signal in the whole brain (*N* = 1), head motion exceeding 3 mm in translation or 3 degrees in rotation (*N* = 9) (Figure 1). Finally, 73 MTLE patients were included in further analysis. Further, MTLE patients who had 2 years follow-up visit after anterior temporal lobectomy (ATL) surgery without any other resection beyond temporal lobe were selected for subsequent surgical outcome prediction analysis. Outcomes were evaluated by the Engel Epilepsy Surgery Outcome Scale (Engel, 1993) by an epileptologist, in which patients with Engel class I were considered seizure-free and subjects with Engel class II–IV were regarded as non-seizure-free. Inclusion criteria for healthy controls include (1) free of psychiatric or neurologic disorders; (2) no contraindications for MRI or PET scanning.

### 2.2. Data acquisition

All participants were scanned on a hybrid PET/MR scanner (Biograph mMR; Siemens Healthcare, Erlangen, Germany) using an 8-channel phase-array head coil. Note that, all patients discontinued anti-epileptic medicine for at least 24 h, and fast for 4–6 h, before the PET/MR scan. T1-weighted structural images were obtained employing a magnetization-prepared rapid gradient echo sequence (echo time = 2.44 ms, repetition time = 1,900 ms, flip angle = 9°, field of view = 250 mm × 250 mm, 192 slices per slab, slice thickness = 1.0 mm, gap = 0.5 mm, matrix = 256 × 256). T2-weighted FLAIR images were acquired with the following parameters: echo time = 92 ms, repetition time = 8,460 ms, flip angle = 150°, field of view = 220 mm × 220 mm, 45 slices per slab, slice thickness = 3.0 mm, gap = 0.4 mm, matrix = 256 × 256. Resting-state fMRI images were acquired using echo-planar imaging sequence (echo time = 30 ms, repetition time = 3,000 ms, flip angle = 90°, field of view = 192 mm × 192 mm, axial slices = 35, slice thickness = 3 mm, gap = 0.8 mm, matrix = 64 × 64, 200 time points). FDG PET images were obtained at 30–50 min post a bolus injection of 3.7 MBq/kg of ^18^F-FDG with the following parameters: 128 slices per slab, gap = 0.5 mm; matrix size = 344 × 344, reconstructed with 3D OSEM iterative reconstruction (subsets = 21, iterations = 4) and post-filtered with an isotropic full-width half-maximum (FWHM) Gaussian kernel of 2 mm.

### 2.3. Data preprocessing

The individual Desikan-Killiany parcellation and thalamic nuclei segmentations were obtained from T1-MPRAGE using FreeSurfer v7.0 package (Desikan et al., 2006; Iglesias et al., 2018), and individual T1-weighted images were resliced to an isotropic voxel size of 2 × 2 × 2 mm^3^. ^18^F-FDG PET images were preprocessed using the package of SPM12 (http://www.fil.ion.ucl.ac.uk/spm/software/spm12) in Matlab (R2019a). The images were co-registered to individual T1-weighted images. In order to reduce inter-subject variance before group analysis, registered FDG PET images were further normalized by cerebellar scaling to obtain SUVR images (Boscolo Galazzo et al., 2016; Lamarche et al., 2016; Wang et al., 2020), assuming little metabolic alterations in cerebellum in MTLE patients. Preprocessed PET images were further smoothed using a FWHM Gaussian kernel of 8 mm for DMN region analysis and 4 mm for subthalamic nuclei analysis.

The resting-state fMRI data were preprocessed using SPM12 and DPARSFA toolbox (Chao-Gan and Yu-Feng, 2010). After removing the first 10 volumes, the remaining fMRI images were realigned and slice-timing corrected. Subsequently, six head motion parameters, white matter and cerebrospinal fluid mean signals were regressed out as nuisance variables (Zuo et al., 2013). In addition, to take into account signal drifts that arise from scanner instability, linear trend was analogously removed from each voxel's time course. The images were then co-registered to individual T1-weighted images and smoothed using FWHM Gaussian kernels of 8 mm and 4 mm that matched the kernels for FDG PET. fALFF maps, which measures the relative contribution of specific low frequency range (0.01–0.08 HZ) oscillations to the whole frequency range of signal variations (Zou et al., 2008), were finally calculated.

The brain regions in Desikan-Killiany atlas were used to define DMN consisting of 28 brain regions according to previous studies (Desikan et al., 2006; Buckner et al., 2008; Andrews-Hanna et al., 2010; Kabbara et al., 2017; Buckner and DiNicola, 2019), and bilateral thalamus. We also segmented the DMN and thalamus in to 6 modules, as shown in Table 1. Based on the thalamic nuclei segmentations, we merged some small nuclei and then divided the thalamus into six large nuclei (see Table 2). Mean SUVR and fALFF of each region-of-interest (ROI) were extracted for subsequent analyses.

**Table 1:** Desikan-Killiany atlas structures forming the DMN and thalamus.

**Region**	**Abbreviation**
**Module 1. Medial temporal lobe**	
Hippocampus	HIP
Parahippocampal gyrus	PHG
**Module 2. Temporal cortex**	
Inferior temporal gyrus	ITG
Middle temporal gyrus	MTG
Superior temporal gyrus	STG
Temporal pole	TPO
**Module 3. Dorsal and ventral medial prefrontal cortex**	
Lateral orbitofrontal	LOF
Medial orbitofrontal	MOF
Middle frontal gyrus	MFG
Isthmus cingulate gyrus	ICG
Anterior cingulate gyrus	ACG
**Module 4. Precuneus/posterior cingulate cortex**	
Posterior cingulate gyrus	PCG
Precuneus	PCUN
**Module5. Inferior parietal lobe**	
Inferior parietal cortex	IPC
**Module 6. Thalamus**	
Thalamus	THA

**Table 2:** Six thalamic nuclei generated from individual T1-weighted images segmentations.

**Region**	**Abbreviation**
**1. Anterior thalamic nuclei**	THA_Anterior
Anteroventral	THA_AV
**2. Lateral thalamic nuclei**	THA_Lateral
Laterodorsal	THA_LD
Lateral posterior	THA_LP
**3. Ventral thalamic nuclei**	THA_Ventral
Ventral posterolateral	THA_VPL
Ventral lateral anterior	THA_VLa
Ventral magnocellular	THA_VAmc
Ventral anterior	THA_VA
Ventromedial	THA_VM
Ventral lateral posterior	THA_VLp
**4. Intralaminar thalamic nuclei**	THA_Intralaminar
Centromedian	THA_CM
Parafascicular	THA_Pf
Central medial	THA_CeM
Central lateral	THA_CL
**5. Medial thalamic nuclei**	THA_Medial
Mediodorsal medial magnocellular	THA_MDm
Mediodorsal lateral parvocellular	THA_MDl
Medial ventral	THA_MV(Re)
**6. Posterior thalamic nuclei**	THA_Posterior
Lateral geniculate	THA_LGN
Medial geniculate	THA_MGN
Pulvinar inferior	THA_PuI
Pulvinar medial	THA_PuM
Suprageniculate	THA_L-Sg
Pulvinar anterior	THA_PuA
Pulvinar lateral	THA_PuL

### 2.4. Network construction and graph theoretical analysis

The metabolic covariance network and functional network were constructed based on interregional metabolic correlation matrices and FC matrices, respectively. The nodes in two modality networks are both defined as 28 brain structures forming DMN plus bilateral thalamus. The interregional metabolic correlations across subjects between the SUVR of every pair of regions for each group, and FC between regions for each participant were calculated by the Pearson correlation coefficient as edges on networks. Negative metabolic and functional correlations were eliminated from further analysis (Tzourio-Mazoyer et al., 2002; Wang et al., 2015). We applied the network density range to the adjacent matrix to ensure the brain network of every subject contained the same number of sides (Xu et al., 2019), thus we constructed networks via a network density range of 5%-50% at an interval of 0.01 in concordance with another study (He et al., 2017).

Based on the constructed brain network, the topological network characteristics, BC was computed to describe the hubness of brain structures forming DMN and thalamus in MTLE patients. Nodes with high BC play the role of a gatekeeper to connect the nodes and sub-groups, so they can most frequently control information flows in the network (Burt, 2018). More precisely, BC of node k (i.e., *p*_*k*_) is formulated as follows:


$$BC\left(p_{k}\right)=\sum_{i<j}^{n}\frac{g_{ij}\left(p_{k}\right)}{g_{ij}};\;i\neq j\neq k$$

where *g*_*ij*_ is the shortest paths linking *p*_*i*_ and *p*_*j*_ and *g*_*ij*_(*p*_*k*_) is the shortest paths linking *p*_*i*_ and *p*_*j*_ that contains *p*_*k*_.

Then BC was calculated at each sparsity threshold, and we applied an area under the curve (AUC) approach to avoid the specific selection of a threshold and provided a summarized scalar for topological network characterization (Zhang et al., 2011; Kim et al., 2017).

### 2.5. Statistical analysis

Mann-Whitney U tests were used to perform all the group comparisons, and Chi-square was used to check gender difference between patient groups and HC group (using the false discovery rate [FDR] to correct for multiple comparisons). Effects of gender, age were removed using a multiple regression model. For SUVR and fALFF, group comparison was performed between MTLE patients and healthy controls in thalamus and brain regions of DMN. Spatial voxel-wise couplings between SUVR and fALFF were calculated using Spearman correlation coefficient in thalamus and DMN regions, and in thalamic nuclei. Group comparison of SUVR-fALFF couplings were performed between patient groups and HC group in thalamus and brain regions of DMN.

The AUC values of BC in functional network nodes and FC were compared between patient groups and HC group. Metabolic network metrics were validated by a bootstrap approach in the present study, by which 100 group-level metabolic covariance networks were generated for each group (Wang et al., 2019), which relied on random sampling with replacement in statistics (Roels et al., 2015), and between-group difference of BC and metabolic connectivity (MC) in metabolic covariance network were further obtained from 100 random bootstrap simulations. Before assessing the coupling between spontaneous neural activities strength and glucose metabolism, SUVR and fALFF maps were linearly standardized into Z-values by subtracting from the mean value of voxels from the individual's whole-brain mask (created by applying a 50% threshold onto the SPM's priori brain mask), and then dividing this difference by the standard deviation across brain mask voxels. Since both the FDG PET contrast and BOLD signal arise mainly from gray matter, to assess as much as possible the “pure” correlation between SUVR and fALFF maps, partial correlation analysis was performed, in which the gray matter fraction was regressed out voxel-wise from the Z-scores for each modality. Therefore, partial correlation coefficient was measured as Spearman ρ between the residuals from the linear regression of SUVR and fALFF map, and the GM fraction map.

**Figure 2:**
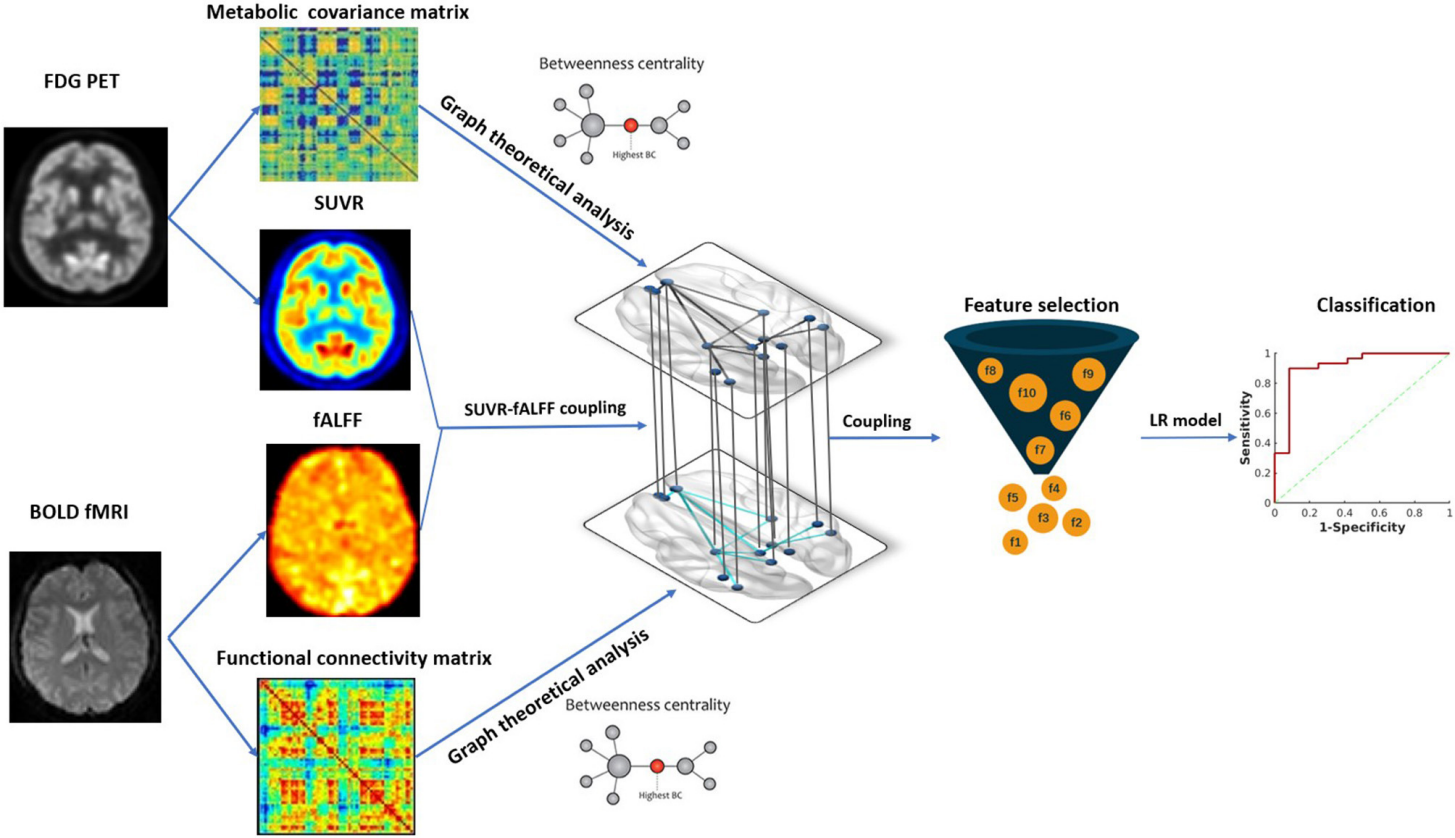
The framework of data analysis. Image features from hybrid FDG PET/fMRI imaging were extracted, including SUVR, fALFF and BC in metabolic and functional network. Metabolic-functional couplings were calculated by SUVR and fALFF map. All the image features and coulings were subsequently compared between MTLE patients and HC. SUVR-fALFF couplings were used for further surgical outcome prediction by Fisher score algorithm for feature selection and logistic regression model for classification. SUVR, standardized uptake value ratio; fALFF, fractional amplitude of low-frequency fluctuation; BC, betweenness centrality; LR, logistic regression.

We further investigated the predictive value of intermodality correlation for surgical outcomes. Firstly, we used Fisher score for feature selection to select the top ten couplings with higher scores as the optimal subset for further analysis (Gu et al., 2012). To evaluate the performance of altered SUVR-fALFF coupling in the surgical outcome prediction, logistic regression model was used to discriminate seizure-free and non-seizure-free groups, with leave-one-out cross-validation. The hyper-parameters of the model were optimized using a gradient search strategy in the training data with Python scikit-learn. The AUC of receiver operating characteristic curve (ROC) was calculated as a quantized measurement of the predictive performance. The data analysis framework of the study is shown in Figure 2.

## 3. Results

### 3.1. Patient demographics

Detailed demographics and clinical characteristics of all participants were listed in Table 3. Thirty-eight patients showed hippocampal sclerosis on routine MR images, and 35 were MR-negative. Of these 30 MTLE patients were seizure-free, 12 MTLE patients were non-seizure-free. Healthy controls were older than patients (*P* < 0.001). Thus, the group comparisons were performed after controlling for age by considering them as covariates.

**Table 3:** Participant demographics.

	**MTLE**		**HC (*n* = 34)**	***P*-value (MR-HS vs HC)**	***P*-value (MR-negative vs HC)**
	**MR-HS (*****n*** = **38)**	**MR-negative (*****n*** = **35)**			
Age (years), median (range)	27 (13-58)	25 (14-54)	46 (32-63)	< 0.001^a^	< 0.001^a^
Gender (M/F)	19/19	23/12	15/19	0.618^b^	0.071^b^
Laterality (L/R)	23/15	21/14	**/**	**/**	**/**
Age at onset (years), median (range)	17 (1-47)	16 (1-53)	**/**	**/**	**/**
Epilepsy duration (years), median (range)	10 (1-43)	5 (1-42)	**/**	**/**	**/**
Seizure frequency (per year), median (range)	24 (1-300)	12 (1-400)	**/**	**/**	**/**
Surgical outcomes^c^ (Engel class I/Engel class II-IV)	17/7	13/5	**/**	**/**	**/**

MTLE, mesial temporal lobe epilepsy; HS, hippocampus sclerosis; HC, healthy controls; a, Mann–Whitney U test; b, Chi-square test; c, surgical outcomes of 24 patients with 2 years follow up after ATL surgery.

### 3.2. Altered glucose uptake and metabolic covariance network properties in MTLE patients

**Figure 3:**
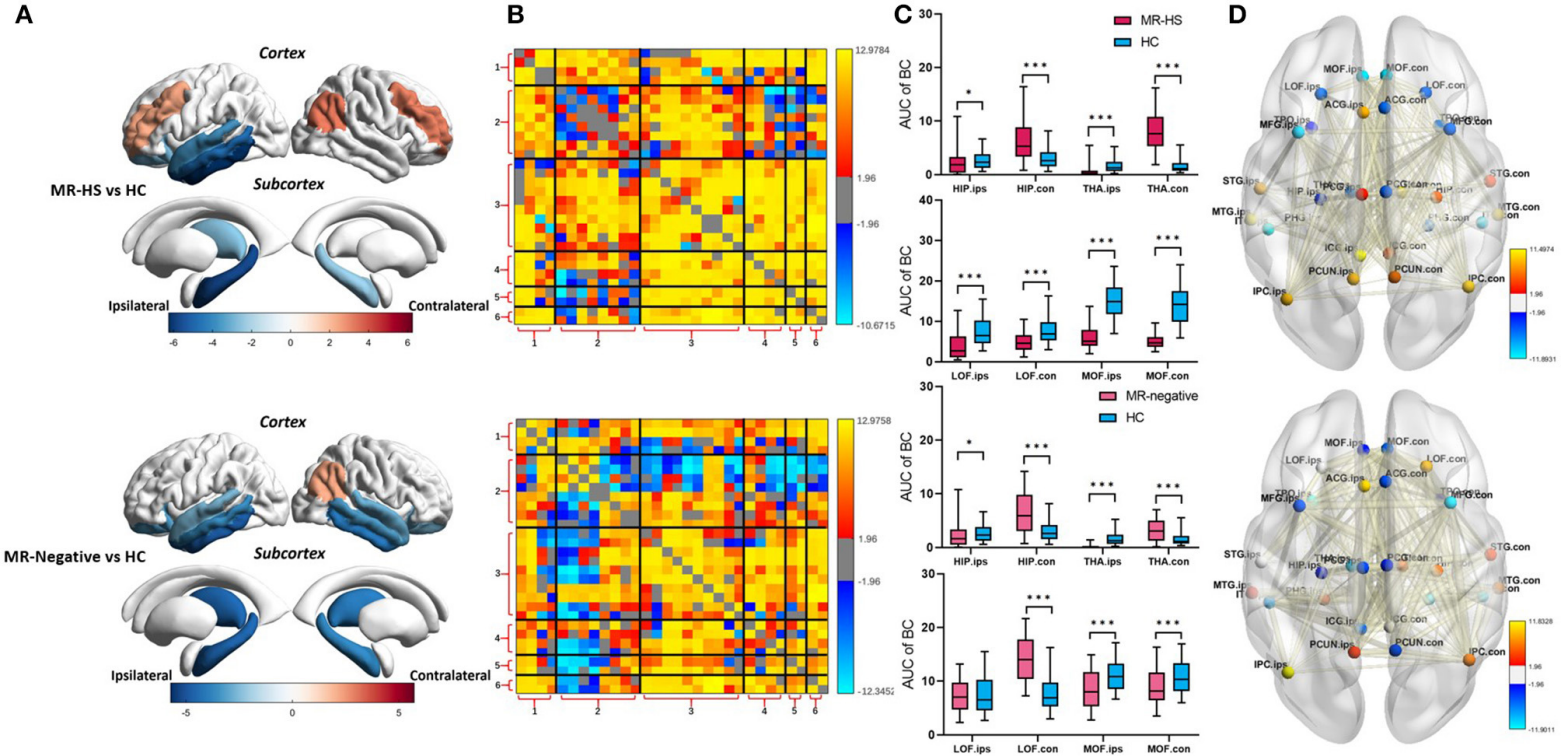
**(A)** SUVR, **(B)** MC, **(C)** BC, and **(D)** BC and MC of brain regions with significant differences in patients, compared with healthy controls. Statistical Z values of significant results of **(A)** SUVR, **(B)** MC, and **(D)** BC and MC were mapped into the corresponding regions or edges with thickness. Warm color represents a significant increase in patient groups, while cold color shows a significantly decreased trend. MR-HS, MTLE patients with hippocampal sclerosis; HC, healthy controls; SUVR, standardized uptake value ratio; BC, betweenness centrality; MC, metabolic connectivity; HIP, hippocampus; PHG, parahippocampal gyrus; ITG, inferior temporal gyrus; MTG, middle temporal gyrus; STG, superior temporal gyrus; TPO, temporal pole; LOF, lateral orbitofrontal; MOF, medial orbitofrontal; MFG, middle frontal gyrus; ICG, isthmus cingulate gyrus; ACG, anterior cingulate gyrus; PCG, posterior cingulate gyrus; PCUN, precuneus; IPC, inferior parietal cortex; THA, thalamus; ips, ipsilateral; ips, ipsilateral; con, contralateral; 1, medial temporal lobe; 2, temporal cortex; 3, dorsal and ventral medial prefrontal cortex; 4, precuneus/posterior cingulate cortex; 5, inferior parietal lobe; 6, thalamus; *P_*FDR*_ < 0.05, **P_*FDR*_ < 0.01, ***P_*FDR*_ < 0.001.

Both MR-HS and MR-negative groups had significantly reduced SUVR in temporal structures, compared with HC. In MR-HS patients, decreased SUVR was mainly located in the ipsilateral hippocampus, thalamus, temporal cortex, lateral orbitofrontal and isthmus cingulate gyrus, whereas higher SUVR was found in the bilateral middle frontal gyrus, contralateral inferior parietal cortex. MR-negative subjects had lower SUVR in the bilateral hippocampus, thalamus, temporal cortex, orbitofrontal, isthmus cingulate gyrus, contralateral medial orbitofrontal and anterior cingulate gyrus than HC, while elevated SUVR was observed in the contralateral inferior parietal cortex (Figure 3A, Supplementary Table 1). MR-HS and MR-negative subjects had a majority of reduced intramodular and intermodular MCs in the module of temporal cortex, while increased intramodular and intermodular MCs were found in other areas of DMN, and MR-HS patients showed more increased MCs than MR-negative patients (Figure 3B). Compared to HC, MR-HS patients showed decreased BC in ipsilateral hippocampus (*P*_*FDR*_ = 0.0152), thalamus (*P*_*FDR*_< 0.001), bilateral lateral orbitofrontal (*P*_*FDR*_< 0.001) and medial orbitofrontal (*P*_*FDR*_< 0.001), and increased BC in contralateral hippocampus (*P*_*FDR*_< 0.001) and thalamus (*P*_*FDR*_< 0.001); MR-negative patients had reduced BC in ipsilateral hippocampus (*P*_*FDR*_ = 0.0125), thalamus (*P*_*FDR*_< 0.001) and bilateral medial orbitofrontal (*P*_*FDR*_ = 0.00046, *P*_*FDR*_< 0.001), and increased BC in contralateral hippocampus (*P*_*FDR*_< 0.001), thalamus (*P*_*FDR*_< 0.001) and lateral orbitofrontal (*P*_*FDR*_< 0.001) (Figure 3C, Supplementary Table 2). Compared with HC group, MR-HS and MR-negative patients showed altered metabolic connectivity and altered Betweenness Centrality (Figure 3D).

### 3.3. Reduced functional activity and altered functional network properties in MTLE patients

**Figure 4:**
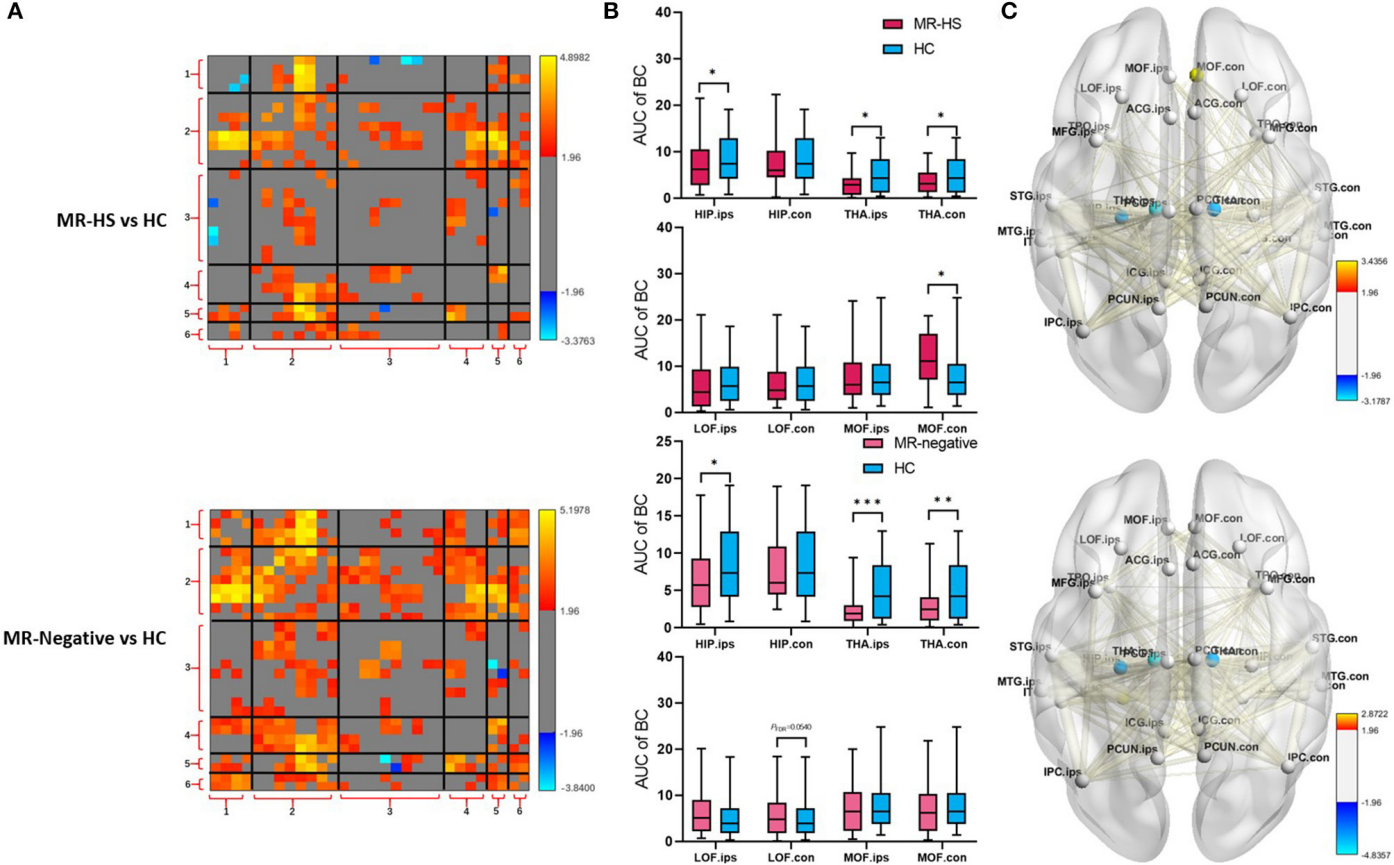
**(A)** FC, **(B)** BC, and **(C)** BC and FC of brain regions with significant differences in patients, compared with healthy controls. Statistical Z values of significant results of **(A)** FC and **(C)** BC and FC were mapped into the corresponding regions or edges with thickness. Warm color represents a significant increase in patient groups, while cold color shows a significantly decreased trend. MR-HS, MTLE patients with hippocampal sclerosis; HC, healthy controls; fALFF, fractional amplitude of low-frequency fluctuation; BC, betweenness centrality; FC, functional connectivity; HIP, hippocampus; PHG, parahippocampal gyrus; ITG, inferior temporal gyrus; MTG, middle temporal gyrus; STG, superior temporal gyrus; TPO, temporal pole; LOF, lateral orbitofrontal; MOF, medial orbitofrontal; MFG, middle frontal gyrus; ICG, isthmus cingulate gyrus; ACG, anterior cingulate gyrus; PCG, posterior cingulate gyrus; PCUN, precuneus; IPC, inferior parietal cortex; THA, thalamus; ips, ipsilateral; ips, ipsilateral; con, contralateral; 1, medial temporal lobe; 2, temporal cortex; 3, dorsal and ventral medial prefrontal cortex; 4, precuneus/posterior cingulate cortex; 5, inferior parietal lobe; 6, thalamus; *P_*FDR*_ < 0.05, **P_*FDR*_ < 0.01, ***P_*FDR*_ < 0.001.

Reduced fALFF in bilateral middle frontal gyrus were found in MR-negative subjects (*P*_*FDR*_< 0.001) (Supplementary Table 1). MR-HS and MR-negative patients both had increased FCs in most regions of DMN, whereas MR-HS showed reduced FCs between ipsilateral hippocampus and regions in dorsal and ventral medial prefrontal cortex (Figure 4A). Compared to HC, MR-HS patients showed decreased BC in ipsilateral hippocampus (*P*_*FDR*_ = 0.0429), bilateral thalamus (*P*_*FDR*_ = 0.0222, *P*_*FDR*_ = 0.0429) and increased BC in contralateral medial orbitofrontal (*P*_*FDR*_ = 0.0177); MR-negative subjects had reduced BC in ipsilateral hippocampus (*P*_*FDR*_ = 0.0216), bilateral thalamus (*P*_*FDR*_ < 0.001, *P*_*FDR*_ = 0.0013) and marginally decreased BC in contralateral lateral orbitofrontal (*P*_*FDR*_ = 0.0540) (Figure 4B, Supplementary Table 2). Compared to HC group, MR-HS and MR-negative patients showed altered functional connectivity and Betweenness Centrality (Figure 4C).

### 3.4. Altered coupling between SUVR and fALFF in MTLE patients

**Figure 5:**
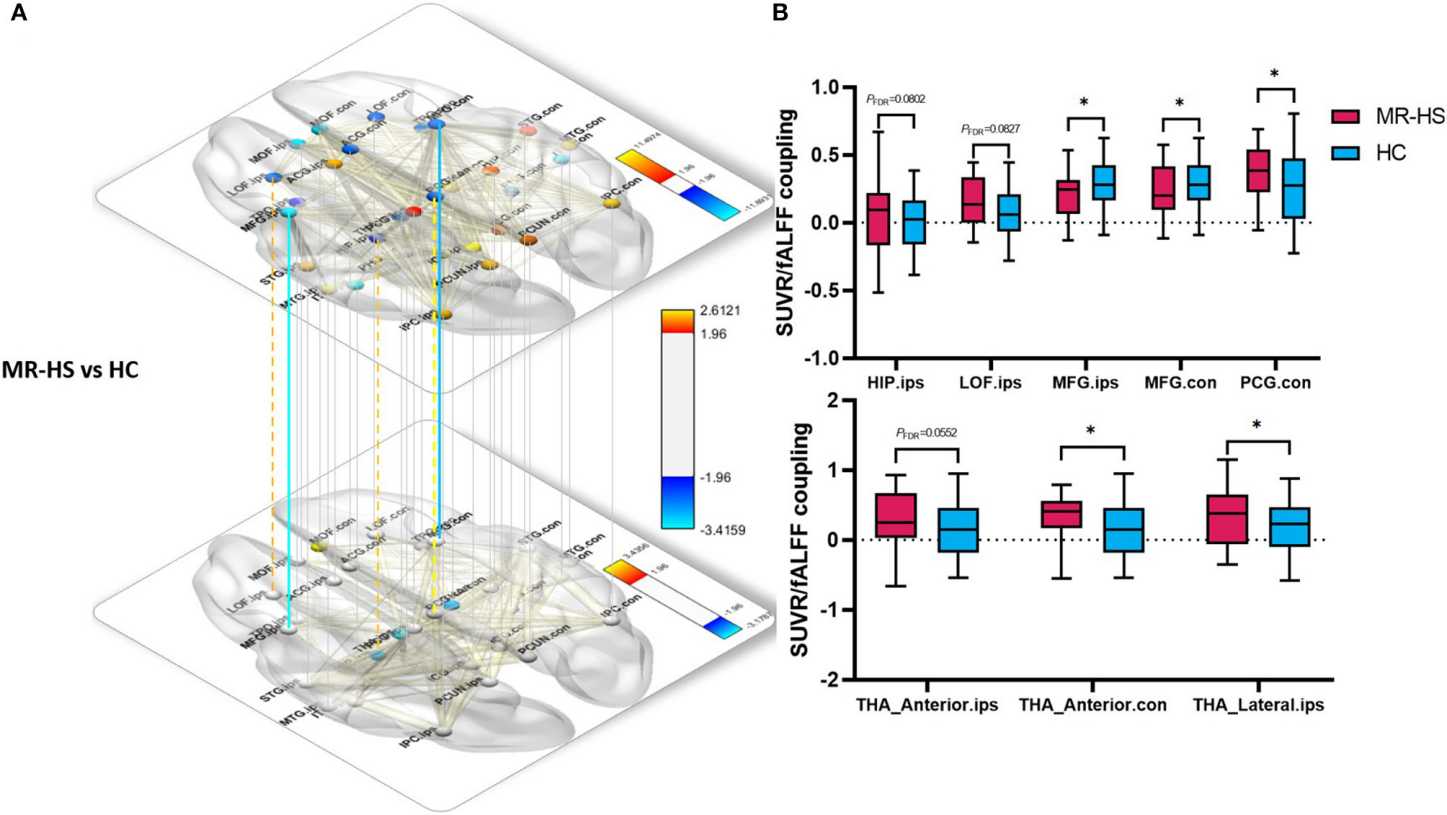
Spatial voxel-wise correlations in regions of DMN thalamic nuclei between SUVR and fALFF with significant group differences between MR-HS patients and HC. **(A, B)** Solid lines represent significantly different SUVR-fALFF couplings, while dotted lines represent marginally different SUVR-fALFF couplings. Warm color represents a significant increase in patient groups, while cold color shows a significantly decreased trend. SUVR, standardized uptake value ratio; fALFF, fractional amplitude of low-frequency fluctuation; MR-HS, MTLE patients with hippocampal sclerosis; HC, healthy controls; ORBmed, medial orbitofrontal cortex; PCUN, precuneus; HIP, hippocampus; LOF, lateral orbitofrontal; MFG, middle frontal gyrus; PCG, posterior cingulate gyrus; THA_Anterior, anterior thalamic nuclei; THA_Lateral, lateral thalamic nuclei; ips, ipsilateral; con, contralateral; *P_*FDR*_ < 0.05, **P_*FDR*_ < 0.01, ***P_*FDR*_ < 0.001.

Regarding metabolic-functional coupling, all significant and marginally significant group differences were mapped into the solid lines and dotted lines between two brain regions nodes, respectively (Figure 5A, Supplementary Table 3). Of note, compared with positive SUVR-fALFF correlations in corresponding brain regions of HC group, MR-HS patients showed marginally elevated coupling in the ipsilateral hippocampus (*P*_*FDR*_ = 0.0802), lateral orbitofrontal (*P*_*FDR*_ = 0.0827) and contralateral posterior cingulate gyrus (*P*_*FDR*_ = 0.0630), whereas decreased coupling in bilateral middle frontal gyrus (*P*_*FDR*_ = 0.0230, *P*_*FDR*_ = 0.0296) were found. Although there was no difference in SUVR-fALFF coupling within thalamus between MR-HS, MR-negative and HC groups, increased coupling in contralateral anterior thalamic nuclei and ipsilateral lateral thalamic nuclei (*P*_*FDR*_ = 0.0230, *P*_*FDR*_ = 0.0470) and marginally increased coupling in ipsilateral anterior thalamic nuclei were obtained in MR-HS patients (*P*_*FDR*_ = 0.0552), compared with HC (Figure 5B, Supplementary Table 3).

### 3.5. Intermodality coupling could help predict surgical outcomes

**Figure 6:**
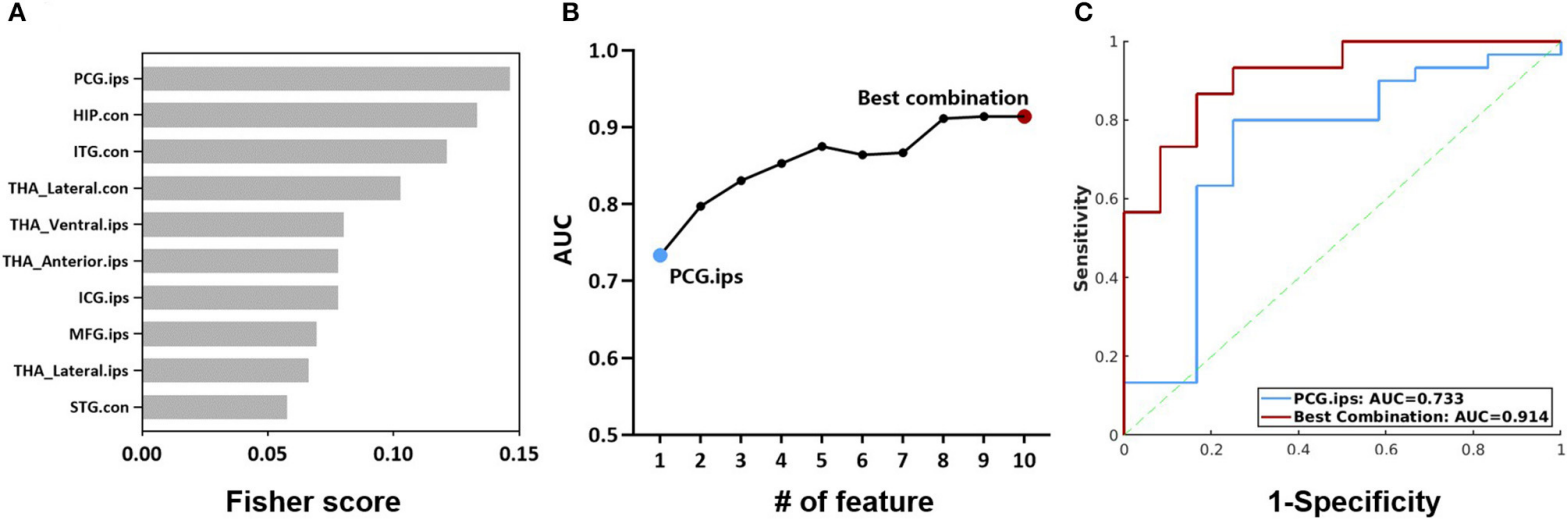
**(A)** Fisher scores of the top 10 SUVR-fALFF couplings. **(B)** AUC of couplings with the top 10 Fisher scores, accumulated from the coupling with the highest Fisher score. **(C)** ROC curves of the classification models with SUVR-fALFF coupling in ipsilateral posterior cingulate gyrus and the best combination. HIP, hippocampus; ITG, inferior temporal gyrus; STG, superior temporal gyrus; MFG, middle frontal gyrus; ICG, isthmus cingulate gyrus; PCG, posterior cingulate gyrus; THA_Anterior, anterior thalamic nuclei; THA_Lateral, lateral thalamic nuclei; THA_Ventral, Ventral thalamic nuclei; ips, ipsilateral; con, contralateral; AUC, the area under ROC curve; SUVR, standardized uptake value ratio; fALFF, fractional amplitude of low-frequency fluctuation.

Further, we evaluated the potential of the intermodality coupling in surgical outcome prediction in 42 MTLE patients with 2 years follow-up visits after ATL surgery. With Fisher score ranking, SUVR-fALFF couplings in regions from thalamic subnuclei such as bilateral lateral thalamic nuclei and ipsilateral anterior thalamic nuclei, regions from DMN, as well as ipsilateral posterior cingulate gyrus, contralateral hippocampus and ipsilateral middle frontal gyrus were selected for the subsequent classification analysis (Figure 6A).

The performances of the combination of SUVR-fALFF couplings from different brain regions were evaluated by logistic regression models, characterized by the ROC curves shown in Figures 6B, C. The top one feature of ipsilateral posterior cingulate gyrus gave a surgical outcome prediction at AUC of 0.733, while the best performance was the combination of ten SUVR-fALFF couplings with the AUC of 0.914 (Figure 6C).

## 4. Discussion

In this study, we investigated whether coupling exist between glucose metabolism and functional activity within the epileptogenic network in MTLE patients using simultaneous ^18^F-FDG PET/rs-fMRI, and examined the predictive value of metabolic-functional couplings in surgical outcome. Specifically, among regions with altered SUVR-fALFF couplings, ipsilateral hippocampus was also decreased in BC of metabolic covariance network and functional network in MR-HS patients. Further, patients with different surgical outcomes were found to have different SUVR-fALFF couplings within the epileptogenic network. Our findings indicated that neuroenergetic coupling measured by PET/MR could improve the comprehending of the pathogenesis in MTLE patients and provide a new neuroimaging metric related to surgical outcome prediction.

Regional hypometabolism could result from repetitive seizures, which is typically characterized by neuronal damage (Patel et al., 2019; Reddy et al., 2019). Chassoux et al. (2016) demonstrated that hypermetabolism was observed in areas partly coincided with DMN, which is in general agreement with the observation of elevated glucose uptake within extratemporal regions of DMN in this study. Hippocampus appears as an altered metabolic hub node in the epileptogenic network as BC decreased in the ipsilateral hippocampus while increased in the contralateral hippocampus. A previous study on metabolic network of MTLE patients found reduced connectivity in ipsilateral hippocampus and increased connectivity in contralateral hippocampus using hippocampus-based metabolic covariant network (Wang et al., 2019). It has been shown that thalamic structural disconnections occur with bilateral mesial temporal lobe and extratemporal regions in MTLE (Barron et al., 2014; Besson et al., 2014), which reveals the structural foundation for metabolic network changes in the thalamus. In addition, decreased connectivities were observed in more brain regions of MR-negative patients compared to MR-HS group, which might suggest a wider epileptogenic network in MR-negative group (Muhlhofer et al., 2017).

In line with previous study, decreased fALFF was also found in medial frontal gyrus in MR-negative patients, which might be explained that the function of DMN was impaired in MTLE subjects and the impairment was attributed to the seizures generation (Zhang et al., 2010). A previous study suggested that impairment of FCs of the affected hippocampus could relate to the anatomic injury of this region with the findings that functional and structural connectivity between mesial temporal lobe structures and other DMN regions are disturbed in patients with MTLE (Liao et al., 2011).

The reason for the elevated coupling between glucose uptake and neural activity remains incompletely understood. Correlations between fALFF and SUVR is generally interpreted as synergy of synaptic currents and action potentials and metabolic demands (Leithner and Royl, 2014; Aiello et al., 2015). In epileptic lesion and epileptogenic network, it is conceivable that modulated by neurovascular coupling both neuronal activity and energy consumption go through great increase, during ictal discharges. During interictal period, altered coupling between metabolism and blood supply has also been reported within the epileptic brain (Song et al., 2016). Ipsilateral hippocampus has been reported to show pathological evidence as a result of excessive oxidative stress even neuronal damage (Engel, 2001), whose marginally increased SUVR-fALFF coupling could be pathology driven. In addition, higher SUVR-fALFF coupling in the ipsilateral posterior cingulate gyrus, one of the functional cores of DMN (Liao et al., 2011), may also be driven by pathological origin, confirming MTLE as a network-based disease. On the other hand, bilateral middle frontal gyrus showed reduced SUVR-fALFF coupling which might be explained by neurovascular decoupling. In this case, the mechanism would prevent blood flow from being effectively stimulated by the abnormal neuronal activity at epileptic discharges (Nugent et al., 2015).

The thalamus is a complex brain structure consisting of multiple nuclei having different connectivity with multiple brain regions (Kumar et al., 2017). In this study, alterations in metabolic-functional coupling were not observed in thalamus as a whole. MR-HS group tends to have elevated coupling in bilateral anterior thalamic nuclei and ipsilateral lateral thalamic nuclei. Anterior nucleus of the thalamus is a crucial component of Papez circuit, an important neural circuit for the generation and propagation of limbic seizures in MTLE (Ferreira et al., 2020), and has become a popular target of deep brain stimulation to treat drug-refractory epilepsy (Jankowski et al., 2013). A memory fMRI and probabilistic diffusion tractography study demonstrated that MR-HS patients with pronounced enhancement of connections between ipsilateral lateral thalamic nuclei and epileptic hippocampus, which might ultimately contribute to the preferred seizures propagation pathway (Dinkelacker et al., 2015).

Using the Fisher score ranking, we pinpointed brain regions within the DMN and thalamus where the coupling between SUVR and fALFF contributes to the prediction of surgical outcomes. Especially, the combination of the top ten gave the best performance for prediction. Notably, these regions do not include often resected regions such as ipsilateral hippocampus, amygdala, but are most likely involved in seizure propagation (Chabardes et al., 2005; Bertram et al., 2008; Li et al., 2016; Abel et al., 2018). The mechanism behind how changed metabolic-functional couplings contribute to the seizure recurrence post-surgery merits further investigation.

This study has some limitations. Firstly, healthy controls are not age-matched with MTLE patients, mainly due to the difficulty of recruiting young healthy volunteers in a full protocol with radioactive tracers. Previous studies have demonstrated that older adults had lower glucose uptake in several DMN regions, like frontal lobe, temporal lobe and cingulate, and they showed higher glucose uptake in thalamus (Nugent et al., 2015; Bonte et al., 2017). In addition, average functional connectivity within resting state networks has been reported to decrease with age (Betzel et al., 2014). In this study, we used multiple regression models for age correction to mitigate the effect of age differences between patients and healthy controls on the results. Secondly, in this preliminary study the sample size for surgical outcome prediction is relatively small, though we used leave-one-out cross-validation to test the classification models, further independent datasets are needed to cross validate the findings. Finally, the complex interactions between functional activity and metabolic consumption is incompletely represented by Tomasi et al. (2013). Potentially non-linear models could be constructed to capture the associations in future studies.

## 5. Conclusions

In summary, our study identified altered coupling between glucose metabolism and functional activity within epileptogenic network in patients with MTLE by simultaneous ^18^F-FDG PET/rs-fMRI and its predictive value in surgical outcomes. These findings may provide potentially useful markers for MTLE patients in their preoperative evaluation.

## Data availability statement

The raw data supporting the conclusions of this article will be made available by the authors, without undue reservation.

## Ethics statement

The studies involving human participants were reviewed and approved by Internal Review Board of Ruijin Hospital. Written informed consent to participate in this study was provided by the participants' legal guardian/next of kin.

## Author contributions

SY: conceive design, experimental design, data analysis, figures organization, and manuscript writing. HH: experimental design and manuscript writing. BC: methodology and manuscript review and editing. JLi: investigation, manuscript review, and editing. MZ: data collection and curation. JLu: conceive design, manuscript review and editing, and manuscript writing. All authors contributed to the article and approved the submitted version.
