# Supplementary material for: Altered metabolic-functional coupling in the epileptogenic network could predict surgical outcomes of mesial temporal lobe epilepsy
Source: Front Neurosci. 2023 Jun 8;17:1165982. doi: 10.3389/fnins.2023.1165982 (PMC10286900; doi:10.3389/fnins.2023.1165982)
Supplement: Supplementary file 1 [file Data_Sheet_1.docx]

**Supplementary Table 1 |** Significant differences in SUVR and fALFF between MR-HS, MR-negative and HC

| Region | SUVR | | fALFF | |
| --- | --- | --- | --- | --- |
|  | *P_FDR_* value  (MR-HS vs HC) | *P_FDR_* value  (MR-negative vs HC) | *P_FDR_* value  (MR-HS vs HC) | *P_FDR_* value  (MR-negative vs HC) |
| HIP.ips | <0.001******* | <0.001******* | 0.4846 | 0.6295 |
| HIP.con | 0.0303***** | <0.001******* | 0.5925 | 0.4383 |
| PHG.ips | <0.001******* | <0.001******* | 0.4846 | 0.6295 |
| PHG.con | 0.0234***** | <0.001******* | 0.6094 | 0.6295 |
| ITG.ips | <0.001******* | <0.001******* | 0.2946 | 0.6295 |
| ITG.con | 0.0609 | <0.001******* | 0.6930 | 0.6295 |
| MTG.ips | <0.001******* | 0.0012****** | 0.6930 | 0.8104 |
| MTG.con | 0.7752 | 0.0030****** | 0.4846 | 0.8104 |
| STG.ips | 0.0014****** | 0.0213***** | 0.8562 | 0.8104 |
| STG.con | 0.6666 | 0.0111***** | 0.8215 | 0.5474 |
| TPO.ips | <0.001******* | <0.001******* | 0.3376 | 0.9306 |
| TPO.con | 0.2369 | 0.0100***** | 0.8562 | 0.9062 |
| LOF.ips | 0.0140***** | 0.0045****** | 0.4846 | 0.6295 |
| LOF.con | 0.7752 | 0.0213***** | 0.8004 | 0.6295 |
| MOF.con | 0.6403 | 0.0477***** | 0.8215 | 0.8262 |
| MFG.ips | 0.0303***** | 0.6311 | 0.2964 | <0.001******* |
| MFG.con | <0.001******* | <0.001******* | 0.9921 | <0.001******* |
| ICG.ips | 0.0165***** | 0.0385***** | 0.6930 | 0.6295 |
| ICG.con | 0.2922 | 0.0213***** | 0.6930 | 0.8104 |
| ACG.con | 0.0895 | 0.0285***** | 0.3376 | 0.8104 |
| IPC.con | 0.0035****** | 0.0363***** | 0.8215 | 0.8576 |
| THA.ips | 0.0187***** | <0.001******* | 0.8562 | 0.9204 |
| THA.con | 0.1397 | <0.001******* | 0.8215 | 0.6295 |

MR-HS, MTLE patients with hippocampal sclerosis; HC, healthy controls; SUVR, standardized uptake value ratio; fALFF, fractional amplitude of low-frequency fluctuation; HIP, hippocampus; PHG, parahippocampal gyrus; ITG, inferior temporal gyrus; MTG, middle temporal gyrus; STG, superior temporal gyrus; TPO, temporal pole; LOF, lateral orbitofrontal; MOF, medial orbitofrontal; MFG, middle frontal gyrus; ICG, isthmus cingulate gyrus; ACG, anterior cingulate gyrus; IPC, inferior parietal cortex; THA, thalamus; ips, ipsilateral; con, contralateral; **P_FDR_* < 0.05, ***P_FDR_* < 0.01, ****P_FDR_* < 0.001.

**Supplementary Table 2 |** Significant differences in BC of metabolic covariance network and functional network between MR-HS, MR-negative and HC

| Region | BC of metabolic covariance network | | BC of functional network | |
| --- | --- | --- | --- | --- |
|  | *P_FDR_* value  (MR-HS vs HC) | *P_FDR_* value  (MR-negative vs HC) | *P_FDR_* value  (MR-HS vs HC) | *P_FDR_* value  (MR-negative vs HC) |
| HIP.ips | 0.0152***** | 0.0125***** | 0.0429***** | 0.0216***** |
| HIP.con | <0.001******* | <0.001******* | 0.9711 | 0.6544 |
| PHG.ips | 0.0059****** | <0.001******* | 0.0565 | 0.0291***** |
| PHG.con | <0.001******* | <0.001******* | 0.5232 | 0.8920 |
| ITG.ips | <0.001******* | <0.001******* | 0.8522 | 0.5218 |
| ITG.con | <0.001******* | <0.001******* | 0.6301 | 0.6544 |
| MTG.ips | <0.001******* | 0.0059****** | 0.9711 | 0.8912 |
| MTG.con | <0.001******* | <0.001******* | 0.9711 | 0.8912 |
| STG.ips | <0.001******* | 0.8173 | 0.5118 | 0.8912 |
| STG.con | <0.001******* | <0.001******* | 0.9007 | 0.8435 |
| TPO.ips | 0.1194 | <0.001******* | 0.0605 | 0.0346***** |
| TPO.con | <0.001******* | 0.0103***** | 0.0565 | 0.0626 |
| LOF.ips | <0.001******* | 0.8732 | 0.6222 | 0.2351 |
| LOF.con | <0.001******* | <0.001******* | 0.9711 | 0.0540 |
| MOF.ips | <0.001******* | 0.0046****** | 0.1800 | 0.8920 |
| MOF.con | <0.001******* | <0.001******* | 0.0177***** | 0.1601 |
| MFG.ips | <0.001******* | <0.001******* | 0.1404 | 0.1405 |
| MFG.con | <0.001******* | <0.001******* | 0.9711 | 0.8912 |
| ICG.ips | <0.001******* | <0.001******* | 0.7940 | 0.8912 |
| ICG.con | <0.001******* | 0.9698 | 0.9711 | 0.0291***** |
| ACG.ips | <0.001******* | <0.001******* | 0.2484 | 0.8912 |
| ACG.con | <0.001******* | <0.001******* | 0.9007 | 0.8912 |
| PCG.ips | <0.001******* | <0.001******* | 0.9709 | 0.3952 |
| PCG.con | <0.001******* | <0.001******* | 0.9007 | 0.8912 |
| IPC.ips | <0.001******* | <0.001******* | 0.1051 | 0.0346***** |
| IPC.con | <0.001******* | <0.001******* | 0.9709 | 0.7016 |
| THA.ips | <0.001******* | <0.001******* | 0.9709 | 0.8912 |
| THA.con | <0.001******* | <0.001******* | 0.9711 | 0.8912 |

MR-HS, MTLE patients with hippocampal sclerosis; HC, healthy controls; BC, betweenness centrality; HIP, hippocampus; PHG, parahippocampal gyrus; ITG, inferior temporal gyrus; MTG, middle temporal gyrus; STG, superior temporal gyrus; TPO, temporal pole; LOF, lateral orbitofrontal; MOF, medial orbitofrontal; MFG, middle frontal gyrus; ICG, isthmus cingulate gyrus; ACG, anterior cingulate gyrus; PCG, posterior cingulate gyrus; PCUN, precuneus; IPC, inferior parietal cortex; THA, thalamus; ips, ipsilateral; con, contralateral; **P_FDR_* < 0.05, ***P_FDR_* < 0.01, ****P_FDR_* < 0.001.

**Supplementary Table 3 |** Significant and marginally significant differences in SUVR-fALFF coupling between MR-HS and HC

| Region | *P_FDR_* value (MR-HS vs HC) |
| --- | --- |
| HIP.ips | 0.0802 |
| LOF.ips | 0.0827 |
| MFG.ips | 0.0230***** |
| MFG.con | 0.0296***** |
| PCG.con | 0.0630 |
| THA_Anteior.ips | 0.0552 |
| THA_Anteior.con | 0.0230***** |
| THA_Lateral.ips | 0.0470***** |

MR-HS, MTLE patients with hippocampal sclerosis; HC, healthy controls; SUVR, standardized uptake value ratio; fALFF, fractional amplitude of low-frequency fluctuation; HIP, hippocampus; LOF, lateral orbitofrontal; MFG, middle frontal gyrus; PCG, posterior cingulate gyrus; THA_Anterior, anterior thalamic nuclei; THA_Lateral, lateral thalamic nuclei; ips, ipsilateral; con, contralateral; **P_FDR_* < 0.05, ***P_FDR_* < 0.01, ****P_FDR_* < 0.001.
